# Policy Perspective: Ensuring Comprehensive Care and Support for Gender Nonconforming Children and Adolescents

**DOI:** 10.1089/trgh.2016.0002

**Published:** 2016-05-01

**Authors:** Nadia Dowshen, Rachel Meadows, Maureen Byrnes, Linda Hawkins, Jennifer Eder, Kathleen Noonan

**Affiliations:** ^1^ Craig-Dalsimer Division of Adolescent Medicine, Children's Hospital of Philadelphia, Philadelphia, Pennsylvania.; ^2^ PolicyLab, Children's Hospital of Philadelphia, Philadelphia, Pennsylvania.; ^3^ Department of Pediatrics, Perelman School of Medicine, University of Pennsylvania, Philadelphia, Pennsylvania.; ^4^ Department of Social Work and Family Services, Children's Hospital of Philadelphia, Philadelphia, Pennsylvania.

**Keywords:** gender nonconforming children and adolescents, healthcare policy, healthcare systems, lesbian, gay, bisexual, and transgender (LGBT) youth, transgender

## Abstract

Despite recent notable advances in societal equality for lesbian, gay, bisexual, and transgender (LGBT) individuals, youth who identify as trans* or gender nonconforming, in particular, continue to experience significant challenges accessing the services they need to grow into healthy adults. This policy perspective first offers background information describing this population, their unique healthcare needs, and obstacles when seeking care, including case study examples. The authors then provide recommendations for medical education, health systems, and insurance payers, as well as recommendations for school systems and broader public policy changes to improve the health and well-being of gender nonconforming youth.

## Introduction

Despite notable advances in societal equality for lesbian, gay, bisexual, and transgender (LGBT) individuals in the recent past, LGBT youth often continue to experience significant challenges accessing the services they need to grow into healthy adults. These obstacles can be particularly difficult for youth who identify as trans* or gender nonconforming.

This policy perspective provides information and recommendations for practitioners, administrators, and policymakers to ensure comprehensive care and support and improve health and well-being for gender nonconforming children and adolescents. Although the authors of this perspective work within the healthcare environment, the recommendations are broader since the healthcare system will not achieve these recommendations on its own.

This perspective has four sections: The first section—Background—offers definitions and context related to care for gender nonconforming children and adolescents. The second section—Improving the Healthcare System—describes the unique healthcare needs of gender nonconforming youth and the obstacles encountered when seeking care. This section also proposes recommendations for medical education, health systems, and public and private insurance payers to improve healthcare for gender nonconforming youth. The third section—Going Beyond Healthcare—argues that improving the health of gender nonconforming youth will require actions beyond the healthcare system and proposes recommendations for school systems as well as broader public policy changes. The last section—Conclusion—provides a final summary and additional resources.

**FIG. 1.**
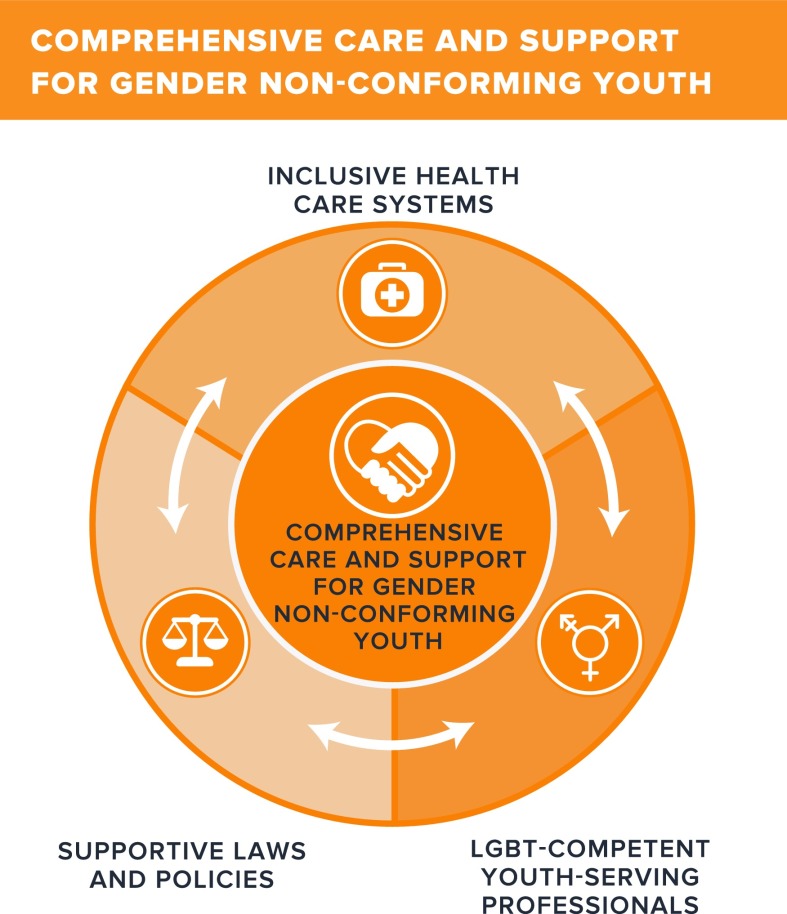
Comprehensive care and support for gender nonconforming youth.

Figure 1 demonstrates how inclusive healthcare systems, supportive laws and policies, and LGBT-competent youth-serving professionals can intersect to ensure comprehensive care and support for gender nonconforming youth.

## Background

### Gender and sexuality

Biological sex refers to a person's physical body and genetic composition and is typically assigned at birth as male or female based on visible anatomy. A person's gender is composed of one's identity, expression, and how these concepts relate to the traditional gender roles and norms of male and female in our society. Gender identity is the gender a person feels and explains their lived experience—who they are. Gender expression is how a person signals their gender identity to the world, for example, the clothes they wear or mannerisms they use.

For most children, biological sex, gender identity, and gender expression are aligned in the way that our society expects. This type of gender expression is called “cis-gender.” However, for some children, their gender identity and/or expression may be different from the sex on their birth certificate. These children are “gender variant,” “gender nonconforming,” or “trans*.” Trans* is not the same as transgender, which is when one's gender identity or gender expression does not match one's assigned sex. Trans* is a more inclusive term than transgender and more deeply acknowledges the spectrum of gender identity and expression. These gender concepts are distinct from sexual orientation, which is who one is attracted to; people who are trans* can have any sexual orientation.

**FIG. 2.**
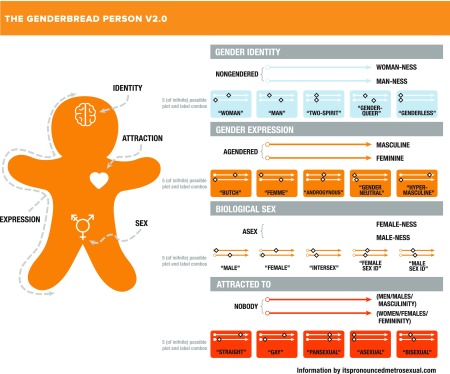
The genderbread person V2.0.

Figure 2 depicts the various spectra of gender identity, gender expression, biological sex, and attraction.

### Gender conformity/nonconformity

Gender conformity is behavior and appearance that conform to societal expectations of one's gender. For example, gender-conforming women may behave and appear in ways that are considered feminine, such as having a long hair style, wearing dresses, and sitting with legs crossed, while gender-conforming men may behave and appear in ways that are considered masculine, such as having short hair and a beard, and wearing pants. Gender nonconformity refers to behavior, identity, or appearance that do not conform to societal expectations of one's gender.

**FIG. 3.**
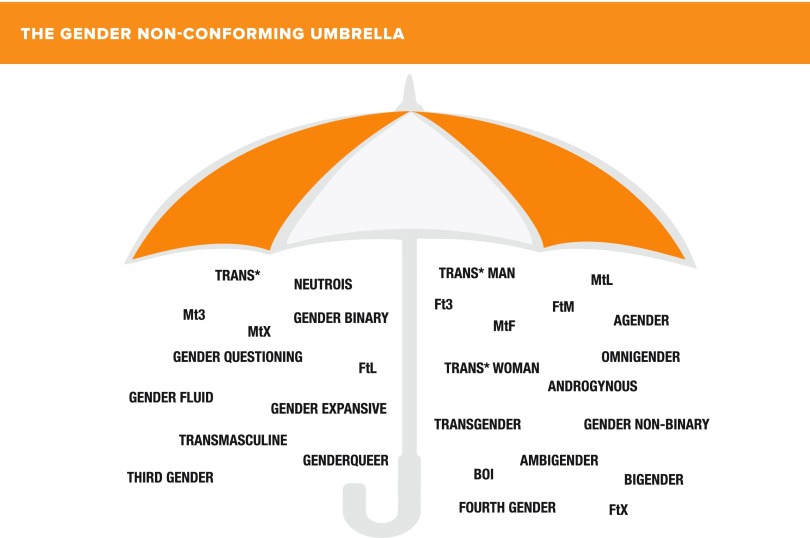
The gender nonconforming umbrella.

We do not know exactly how many people in the world are trans*, gender variant, or gender nonconforming, but data from the National Center for Transgender Equality suggest that the prevalence may be as high as 1% of the general population.^1,2^ Not all children who are trans*, gender variant, or gender nonconforming will grow up to be transgender. This perspective uses the broader, more inclusive umbrella term of gender nonconforming and all that this term includes, as shown in Figure 3.

When gender nonconforming youth experience rejection and isolation, it can have devastating consequences for their physical and emotional health and well-being. Unfortunately, many gender nonconforming youth feel forced to hide their gender identity and expression from others for fear of rejection, bullying, or victimization. The stress and isolation they often face can cause serious psychological distress leading to high levels of anxiety and depression. Rates of suicide attempts are reported to be as high as 40% by the William's Institute at UCLA's School of Law.^3^

Gender nonconforming youth also experience high rates of homelessness, physical violence, and harassment. In addition, because of lack of access to appropriate care, many young transgender women in particular engage in “survival sex” (trading sex for drugs, money, or a place to stay) and use street procedures (e.g., silicone injections, also known as “pumping”), hormones, and other gender-affirming medications not under the supervision of a healthcare provider. Young transgender women also face extremely high rates of HIV infection.^4–9^ There is great need for tailored support and services for this most vulnerable population of gender nonconforming youth who face the world without the support of their families and communities.

## Improving the Healthcare System

Challenges for gender nonconforming youth exist at every turn in our society. They experience a range of reactions from family members, school officials, and classmates, and others in their communities, including a lack of understanding to outright rejection, isolation, discrimination, and victimization. In addition to these societal challenges, there are many barriers in our healthcare system that limit the best health outcomes for gender nonconforming youth. For example, many gender nonconforming youth experience significant obstacles to accessing appropriately trained healthcare providers and needed health and psychosocial services. Also, many gender nonconforming youth are denied insurance coverage for essential health services, such as puberty-blocking medications, cross-gender hormones, and, in some cases, gender reassignment surgery.

The following case studies represent real-life stories of some of the most common challenges experienced by gender nonconforming (GNC) youth and their families and explore potential solutions:

### Case study 1: Jacob/Jackie's story

At 4 years old, Jacob's parents noticed that their son wanted to wear his mother's clothes and wanted to be called “Jackie.” Other kids started making fun of the child at school, and when the child tried to stand in the girls' line or go to the girls' bathroom at school, the child was told “no” by the teachers. The child's parents were concerned about how they should handle this behavior and talked to their pediatrician, who said that it was probably just a phase.

The child's parents decided to ignore the behavior, hoping their pediatrician was right. Over time, the child stopped expressing these feelings in front of others, but began to spend several hours per day doing online gaming, where the child lived and interacted as “Jackie.” The child's parents noticed depressive and increasingly withdrawn behavior as the teenage years began. When the child's parents discovered this other life online, they became very worried and again sought help from their pediatrician, wondering if their child was gay.

#### What went wrong or could have gone better for this child?

Gender identity begins to develop and solidify as early as ages 2–5, so children often begin to express gender nonconformity at this young age.^10^ Parents and teachers are often uncomfortable with this type of behavior and try to discourage or ignore the behavior. Many parents will seek help from a school or health professional in the hope of doing what is best for their child, but oftentimes, these professionals lack training in how to support gender nonconforming youth. Training in this area would go a long way in helping schools and health systems be better prepared to support children, such as Jackie, and their parents.

### Case study 2: Riley's story

Riley was assigned to female sex at birth, but from as early as age 3 insisted that she was a boy. From the time Riley's parents first noted the behavior and as it persisted over the next several years, they supported Riley by using “he/him/his” pronouns. They sought help from their pediatrician, who used Riley's name and preferred pronouns and told him that their practice was a safe place for Riley to be a boy. The pediatrician referred him to a local supportive therapist. The therapist and pediatrician had a discussion with Riley's elementary school about how to be supportive, and Riley was called by his preferred name and pronouns and allowed to use the bathroom of his identified gender.

At age 8, Riley began to see a medical gender specialist at a multidisciplinary clinic, and the decision was made in concert with the clinic's mental health gender specialist and Riley's parents to place a puberty-blocking implant, a fully reversible treatment, at age 10 (as soon as Riley began to develop breast buds). Riley's parents were prepared to pay over $15,000 for this treatment if necessary, because they were told it is often not covered by insurance, even though it is considered the standard of care by World Professional Association for Transgender Health (WPATH) and The Endocrine Society guidelines.^11,12^

The insurance company initially denied payment, but then agreed to reimbursement after several appeals by the multidisciplinary team, which noted that this high up-front cost would be balanced by improved mental health outcomes and less need for costly surgeries later by preventing breast development. Whenever Riley visited the clinic, everyone from the front desk staff to the treating physician to the person who draws blood respected Riley's gender identity by using his preferred name and pronouns, and these preferences were noted in his electronic health record (EHR).

He continued on his medication and persisted in his male gender identity until age 16, when the decision was made collaboratively with his medical and mental health team and parents to start testosterone (a treatment that is considered partially reversible), because they thought Riley was cognitively and socially ready to make such a decision. The clinic social worker also worked with legal advocates to get Riley's gender changed legally (i.e., his gender now says “male” on his driver's license) from a conservative judge who often denied name changes to trans* individuals. The clinic social worker also worked with Riley's middle and high school teachers and administrators to ensure that Riley was called by his preferred name and pronoun by all school staff and fellow students, he could use the bathroom of his choosing, and that he could try out for and play on the sports team of his choosing.

#### What went right for this child, and what challenges remain?

Riley's case shows that positive health and psychosocial outcomes are possible when families, schools, communities, and healthcare providers support a child's gender nonconformity. The struggles for Riley's multidisciplinary care team around obtaining insurance coverage for medications and securing the legal change of his name/gender marker demonstrate some of the key policy and health system challenges that still need to be addressed.

A supportive family, community, and school all play an essential role in creating a healthy developmental support system for gender nonconforming youth. In addition, a knowledgeable and understanding primary care doctor with a basic knowledge of available specialists and treatment options can play an important role in ensuring that gender nonconforming youth receive the health and psychosocial services they need and deserve.

### Recommendations for improving the healthcare system

The following recommendations represent action steps that can be taken to ensure that all physicians who care for children have a basic understanding and set of skills to provide care to gender non-conforming youth. These recommendations also include actions that policymakers can take to address obstacles that stand in the way of ensuring comprehensive care and support, both inside and outside the healthcare setting, for gender nonconforming children and adolescents.

Recommendations:

1. Improve physician education
2. Create and support interdisciplinary treatment teams
3. Support a more gender-inclusive patient care environment
4. Support and expand research
5. Expand insurance coverage for gender-affirming care

1. Improve Physician Education

All medical students should receive a dedicated curriculum and instruction that focus on LGBT health issues and specifically address the medical and psychosocial needs and appropriate treatment of gender nonconforming youth.

In a survey conducted by researchers at The Children's Hospital of Philadelphia (CHOP) and The University of Pennsylvania in 2012, 74% of medical students reported 2 h or less of transgender health instruction during medical school. When gender identity development and trans* health education were included as part of the curriculum for medical students, they reported greater understanding of discrimination and improved knowledge of health outcomes; positive attitudes toward trans*-competent language, resources, and practice environment; improved skill in performing appropriate medical evaluation; and a stronger ability to discuss options for gender-affirming medical therapy, procedures, and devices.^13^

Some medical schools have developed lectures and symposiums on LGBT health issues that specifically address gender nonconforming issues. For example, as a result of the CHOP/University of Pennsylvania survey findings, a 5-h symposium on transgender health was developed at the University of Pennsylvania Medical School that is now part of the curriculum for every medical student at the school. The symposium was initiated by a group of students interested in covering these topics as part of the formal curriculum. The symposium includes a panel of gender nonconforming youth and their parents describing their experiences and interactions with the Gender and Sexuality Development Clinic at CHOP; lectures by an adolescent medicine gender specialist, mental health gender specialist, and urologist who performs gender-affirming procedures; and a provider panel, including primary care and subspecialty providers with experience caring for gender nonconforming patients across their life span.

These kinds of symposiums and training programs should be part of every medical student's educational experience. In fact, the Association of American Medical Colleges (AAMC) recently released a set of guidelines for training physicians to care for people who are lesbian, gay, bisexual, transgender, gender nonconforming, or born with differences of sex development.^14^

Medical training for doctors does not end with medical school and neither should their training in LGBT health. Practicing providers are expected to complete a certain number of Continuing Medical Education (CME) courses each year to maintain their medical license. Transgender health-related CME opportunities should be available to all doctors and should be publicized widely.

Currently, courses that offer trans* health education for CME credit are offered at The Philadelphia Trans-Health Conference, the Gender Spectrum Conference, the Society for Adolescent Health and Medicine, the American Academy of Pediatrics' National Conference & Exhibition, and the Human Rights Campaign's Time to Thrive conference, but this kind of education should not be limited to just these conferences.^15–19^

Courses that offer trans* health education for CME credit should be offered at a wide range of medical society and professional meetings since doctors across all specialties will care for trans* patients. Other nonconference options for obtaining CME credit include webinars, online modules, and other products such as the American Academy of Pediatrics' e-book *Reaching Teens: Strength-Based Communication Strategies to Build Resilience and Support Healthy Adolescent Development*, which includes a chapter on caring for sexual and gender minority youth, and the Fenway Institute's National LGBT Health Education Center's learning module on Caring for LGBTQ Youth in Clinical Settings.^20,21^

2. Create and Support Interdisciplinary Treatment Teams

No single provider can serve all the needs of a gender nonconforming patient. The patient can expect to see several specialists in addition to their primary care provider. Ideally, an interdisciplinary team that meets and communicates on a regular basis should provide care for the youth and their family.

Interdisciplinary teams caring for gender nonconforming children and adolescents should ideally be composed of a supportive primary care doctor; a pediatric medical gender specialist (often an adolescent medicine specialist and/or endocrinologist); a mental health gender specialist; a nurse skilled in care coordination and administering and teaching self-injections, if necessary, for puberty blocking, menstrual suppression, and administration of cross-gender hormones; and a social worker who can advocate for the youth and help the youth access school and other community services. Teams should also have access to surgeons (including urologists, obstetrician/gynecologists, and plastic surgeons) who can provide gender-affirming consultation and procedures when indicated. Organizations such as The WPATH and The Gay and Lesbian Medical Association publish directories where patients and other professionals can find appropriately trained providers in their area.^22,23^

3. Support a More Gender-Inclusive Patient Care Environment

The entire team at a provider's office (including but not limited to front desk staff, billing staff, support staff, and the clinicians) should work together to ensure a patient's experience during the visit is comfortable. The office should have institutional, practice-wide policies and expectations related to acknowledging and respecting a patient's gender expression and identity. The focus should be on training all staff, particularly the front desk, receptionist, and intake staff, who are often the first point of contact and can set the tone for a patient's visit. For example, all staff should be trained on how to best serve a patient whose gender expression and/or identity might not match their current documents (i.e., ID, insurance credentials). All staff should also be aware of spaces that gender nonconforming youth feel most unsafe, such as restrooms and locker rooms, and work to make the practice a safe space.

When it comes to language and communication, it can be challenging and confusing to know how and when to appropriately use terms such as transgender, trans*, gender variant, or gender nonconforming. It can be hard to know which terms are preferred and which terms could be offensive. However, dealing with this issue in a caring and thoughtful way is very important since how we use language validates the identity of many young people. The easiest and most respectful approach is to simply ask the individual in an open and respectful way which term(s) are preferred and what pronoun he/she/they would like you to use.

A major goal of healthcare reform is to improve quality of care through EHRs. The federal guidelines developed to improve the use of EHRs, collectively referred to as Meaningful Use, set standards for provider use of EHRs. EHR systems should have the option to collect sexual orientation/gender identity (SOGI) information and to store information (like preferred names and pronouns) as outlined in the Meaningful Use Stage 3 criteria.^24^ EHR systems should use trans*-affirming language that does not require using only “male” or “female.” However, this information must always be optional to protect adolescent confidentiality.

EHRs can greatly improve patient–provider interactions. For example, when a patient enters a doctor's office and checks in with the front desk, sign-in forms should include an option for preferred pronouns and a “name you would like to be called” option. EHR systems will enable staff to use the patient's preferred name and pronouns, and the patient will not have to explain his/her/their full medical history each time they see a provider.

4. Support and Expand Research

Recent data support the significant benefits of treatments, such as puberty suppression, which are the current standard of care for gender nonconforming youth.^25^ However, more research is needed to understand the optimal timing of specific treatments for gender nonconforming youth with varying medical, mental health, and developmental needs. A better understanding of the prevalence and biologic/genetic underpinnings of varied gender expression and identity will be critical to enhance care options for youth. To take these steps forward, we also need to ensure that researchers are including appropriate questions to collect SOGI information in their studies when relevant and possible as per the recommendations in an Institute of Medicine 2011 report entitled *The Health of Lesbian, Gay, Bisexual, and Transgender People: Building a Foundation for Better Understanding*.^26^ Research must continue to inform practice so that gender nonconforming youth receive the best possible care to develop into happy and healthy adults.

5. Expand Insurance Coverage for Gender-Affirming Care

In July 2012, the U.S. Department of Health and Human Services (HHS) Office of Civil Rights explicitly confirmed that the nondiscriminatory provision in the Affordable Care Act (ACA) prohibits sex discrimination against gender nonconforming people in the health insurance industry. The HHS Office of Civil Rights stated that it would be discriminatory to deny health insurance coverage and benefits based solely on “gender identity or failure to conform to stereotypical notions of masculinity or femininity.”^27^ At the time of writing this perspective, HHS was completing the process of accepting comments on proposed rules for Section 1557 of the ACA that would make it illegal to exclude coverage and services related to gender transition, including gender confirmation surgery, hormone therapy, and counseling.^28^

Despite this strong statement of support from the federal government, gender nonconforming people, including gender nonconforming youth, are frequently denied health insurance coverage for needed healthcare services. For example, only about one-fifth of all the states and the District of Columbia explicitly offer care to transgender individuals, ensuring that transgender residents have access to medically necessary healthcare.^29^

To ensure that health insurance policies do not discriminate against trans* people, the state governor or state insurance commissioner can take action. For example, in a 2014 letter sent to all insurers in New York State, Governor Andrew Cuomo stated, “An issuer of a policy that includes coverage for mental health conditions may not exclude coverage for the diagnosis and treatment of gender dysphoria.”^30^ In Washington State, Insurance Commissioner Mike Kreidler sent a letter to all Washington State insurers in June 2014 clarifying that they must cover transition-related care, such as hormone therapy, counseling services, mastectomy, and breast augmentation and reduction.^29^

New York and Washington can serve as models to other states to ensure that all health insurance policies do not discriminate against trans* people. Furthermore, state policies should also specify that insurers cannot discriminate against needed medical and mental health services specifically for gender nonconforming youth. For example, gender nonconforming youth may require puberty blocking medications/implants, as well as an extended assessment or ongoing therapy with a mental health gender specialist. These products and services may be expensive in the short term, but will likely lead to long-term health benefits, including decreased mortality and morbidity and cost savings. For example, in Riley's story (see Case Study 2), when a gender nonconforming youth who identifies as male starts puberty blockers early, it can prevent any breast development and therefore decrease the need for “top surgery” or a mastectomy later in life, as well as alleviate the psychological distress gender nonconforming youth often face during puberty, both of which will likely lead to better health outcomes and significant cost savings going into adulthood.

## Going Beyond Healthcare

Care for all children does not and should not stop when the child leaves the doctor's office or hospital. Therefore, to ensure comprehensive care for gender nonconforming youth, the focus cannot be just on the healthcare setting. Many gender nonconforming youth experience challenges outside the healthcare setting, including incidents of discrimination and bullying. For example, these youth face challenges at home, in school, within their faith communities, from their community-based organizations, and from social services and child welfare systems. Not addressing these issues can lead to lack of support, rejection, isolation, victimization, and long-term mental health issues for these youth. Addressing these challenges is essential to ensuring that gender nonconforming youth can achieve optimal physical and emotional health outcomes.

### Recommendations for systems outside of healthcare

The following recommendations represent action steps that can be taken to ensure that all gender nonconforming youth grow up in a safe and supportive environment in their schools and communities and have access to a range of legal and social services when needed.

Recommendations

1. Enact state and local policies to ensure protections based on SOGI/expression
2. Extend legal protections for LGBT youth in school
3. Ensure that school districts develop and incorporate LGBT-inclusive curriculum
4. Educate key youth-serving professionals
5. Ensure access to legal and social work supports

1. Enact State and Local Policies to Ensure Protections Based on SOGI/Expression

Only 18 states and the District of Columbia have laws that clearly prohibit discrimination against trans* people when it comes to employment, housing, public accommodations, and/or education. In addition, at least 200 cities have passed legislation that prohibits gender identity discrimination.^31^ Some laws are more robust than others. For example, some laws prohibit employment discrimination, but not housing discrimination, while others prohibit both. The more robust laws explicitly define and/or interpret “sexual orientation” to include gender identity. Policymakers in all cities and states should ensure statutory protections against discrimination that cover gender nonconforming individuals, including gender nonconforming youth.

2. Extend Legal Protections for LGBT Youth in Schools

Only 13 states and the District of Columbia have adopted nondiscrimination laws that apply to schools and protect students on the basis of sexual orientation as well as gender identity.^32^ Laws that ensure protections based on sexual orientation as well as gender identity are necessary, particularly for gender nonconforming youth.

While all 50 states have adopted legislation to address bullying in schools, LGBT protections are not always included in this antibullying legislation.^33^ It is still legal in many parts of the country for school staff to discriminate against LGBT students, and anti-LGBT bullying is still very common in American schools. The Gay, Lesbian, and Straight Education Network (GLSEN), a national organization that focuses on ensuring a safe school for all students, including LGBT students, found that only 18 states and the District of Columbia have “fully enumerated” antibullying laws, which specifically prohibit bullying and harassment of students based on SOGI.^32^ Unfortunately, there are also eight states with laws that expressly forbid teachers from discussing gay and transgender issues (including sexual health and HIV/AIDS awareness and information).^34^ Two additional states prohibit school districts from having enumerated antibullying policies.^32^

At the federal level, two pieces of legislation—the Safe Schools Improvement Act and the Student Non-Discrimination Act—have been introduced as federal antibullying legislation.^35,36^ The Safe Schools Improvement Act would require school districts in states that receive federal funds to adopt codes of conduct that specifically prohibit bullying and harassment on the basis of race, color, national origin, sex, disability, sexual orientation, gender identity, and religion. It would also require that states report data on bullying and harassment to the federal government. The Student Non-Discrimination Act would apply to all schools and specifically addresses the issue of discrimination on the basis of sexual orientation or gender identity. The law would prohibit schools from discriminating against any student on the basis of actual or perceived sexual orientation or gender identity. It would also prohibit schools from discriminating against any student because of the actual or perceived sexual orientation or gender identity of a person with whom that student associates or has associated. The bill would allow an aggrieved individual to assert a violation of the prohibitions in a judicial proceeding. So far, both bills have been considered in a congressional committee, but they have not yet been voted on in the U.S. House of Representatives or Senate.

Some states have adopted legislation that explicitly protects trans* and gender nonconforming students. For example, California's Assembly Bill 1266, enacted in January 2014, gives students in public K-12 schools the right “to participate in sex-segregated programs, activities, and facilities.”^37^ In other words, this bill allows transgender youth to use the bathroom that matches their gender identity and to participate on whichever sports team they believe matches their gender identity. Other states such as Massachusetts, Connecticut, and Washington have statewide policies that ensure such protections, but they are just that—policies—and are not guaranteed by law.^38^ State laws are needed to ensure fair and equal treatment.

3. Ensure That School Districts Develop and Incorporate LGBT-Inclusive Curriculum

Every school district should develop and incorporate LGBT-inclusive curriculum, which, at minimum, allows for a safe school environment by including positive representations of LGBT people in history in the curriculum. Unfortunately, only 18.5% of students from GLSEN's 2013 National School Climate Survey reported being taught positive representations about LGBT people in their schools.^39^ Curricula have been developed to respond to the varied needs of students from differing social, cultural, racial, and ethnic backgrounds, but many curricula still do not incorporate LGBT awareness and education.

An LGBT-inclusive curriculum should use language and examples throughout all content areas that are inclusive of LGBT individuals and do not assume heterosexuality or cis-gender. This is particularly important in health and sex education classes but should apply to all school subjects. The results of a 2009 National School Climate Study conducted by GLSEN reveal that attending a school with an LGBT-inclusive curriculum created a less hostile school experience for LGBT students and also provided increased feelings of “connectedness” to their school communities. In schools with LGBT-inclusive curriculum, students reported that they feel less like victims, are more likely to feel safe, and are less likely to miss school because they feel unsafe or uncomfortable.^40^

While LGBT-inclusive curriculum can be developed at the school district level, some state legislatures have created legislation to ensure that all school districts in the state have LGBT-inclusive curricula. For example, California passed the Fair, Accurate, Inclusive, and Respectful (FAIR) Education Act in July 2011 that ensures LGBT contributions are included in California social science education. The act also prohibits the adoption of textbooks and other instructional materials that discriminate against LGBT people.^41^

4. Educate Key Youth-Serving Professionals

Just as medical students should receive dedicated lesson time focusing on LGBT health issues, so should social workers, teachers, and other key youth-serving professionals. Schools of social work frequently weave LGBT awareness and inclusiveness into the curriculum. However, gender identity development education is not always provided as a formal lecture or symposium. As recommended for medical students, social work students should at the very least receive a lecture and/or symposium during their academic training that focuses on LGBT issues and specifically addresses gender nonconforming youth. In addition, the National Association of Social Workers (NASW) has developed a set of standards for cultural competence in social work practice that should be adopted by all practicing social workers.^42^

Teachers, guidance counselors, and school administrators also play a key role in supporting gender nonconforming youth. In the GLSEN 2013 National School Climate Survey, almost all LGBT students (96%) reported having at least one school staff member whom they believed was supportive of LGBT students at their school. However, 55% of students still reported feeling unsafe due to their sexual orientation and 38% of students reported feeling unsafe due to their gender expression. In addition, half of students attended schools that did not have a Gay-Straight Alliance, and only 10% reported that their schools' antibullying policies specifically enumerated protections based on both SOGI/expression.^39^

Schools should provide appropriate LGBT training to teachers, guidance counselors, school administrators, support staff, bus drivers, and custodial staff. GLSEN provides a free, online guide for such training programs.^43^ Outside of schools, there are many other community-based, social service, child welfare, and youth-serving organizations that interact with LGBT youth, and these organizations need policies and training for working effectively with LGBT youth, especially gender nonconforming youth. In particular, since LGBT youth are disproportionately represented in the juvenile justice system and more likely to be homeless, professionals working in these settings should also receive specific training to identify and support these youth.

5. Ensure Access to Legal and Social Work Supports

Gender nonconforming youth need access to legal and social work supports to legally change their name and gender marker (should they choose) and receive appropriate school and community accommodations and access. For example, these youth may need access to bathrooms, locker rooms, sports/activity teams, and participation in various events that are consistent with their identity, such as father/daughter dances, clubs, prom, and homecoming. The National Center for Transgender Equality is a social justice organization that provides numerous resources to LGBT youth, including a list of organizations that can help youth access legal assistance and support groups.^2^

## Conclusion

Our understanding of gender identity, gender expression, and gender nonconformity has greatly increased in the past several years. Yet, much work remains to ensure environments—including the health system, school, and community—enable gender nonconforming youth to develop and thrive into healthy young adults. The information and recommendations contained in this perspective are intended to help practitioners, administrators, and policymakers understand the needs of gender nonconforming youth and the practices and policies that can contribute to their improved health outcomes. We encourage these decision makers, and any other individuals who work with gender nonconforming youth, to pursue educational and professional resources and advocate for inclusive policies.

## Abbreviations Used

ACA: Affordable Care Act
CHOP: Children's Hospital of Philadelphia
CME: Continuing Medical Education
EHR: electronic health record
GLSEN: Gay, Lesbian, & Straight Education Network
HHS: U.S. Department of Health and Human Services
SOGI: sexual orientation/gender identity
WPATH: World Professional Association for Transgender Health
